# Predicting prognosis in hepatocellular carcinoma after curative surgery with common clinicopathologic parameters

**DOI:** 10.1186/1471-2407-9-389

**Published:** 2009-11-03

**Authors:** Ke Hao, John M Luk, Nikki PY Lee, Mao Mao, Chunsheng Zhang, Mark D Ferguson, John Lamb, Hongyue Dai, Irene O Ng, Pak C Sham, Ronnie TP Poon

**Affiliations:** 1 Rosetta Inpharmatics LLC, Merck Research Laboratories, Seattle, WA, USA; 2 Department of Surgery, The University of Hong Kong, Queen Mary Hospital, Pokfulam, Hong Kong SAR, PR China; 3 Department of Pathology, The University of Hong Kong, Pokfulam, Hong Kong SAR, PR China; 4 Department of Psychiatry and Genome Research Center, The University of Hong Kong, Pokfulam, Hong Kong SAR, PR China; 5 Department of Pharmacology and Department of Surgery, National University Health System, National University of Singapore, Singapore

## Abstract

**Background:**

Surgical resection is one important curative treatment for hepatocellular carcinoma (HCC), but the prognosis following surgery differs substantially and such large variation is mainly unexplained. A review of the literature yields a number of clinicopathologic parameters associated with HCC prognosis. However, the results are not consistent due to lack of systemic approach to establish a prediction model incorporating all these parameters.

**Methods:**

We conducted a retrospective analysis on the common clinicopathologic parameters from a cohort of 572 ethnic Chinese HCC patients who received curative surgery. The cases were randomly divided into training (n = 272) and validation (n = 300) sets. Each parameter was individually tested and the significant parameters were entered into a linear classifier for model building, and the prediction accuracy was assessed in the validation set

**Results:**

Our findings based on the training set data reveal 6 common clinicopathologic parameters (tumor size, number of tumor nodules, tumor stage, venous infiltration status, and serum α-fetoprotein and total albumin levels) that were significantly associated with the overall HCC survival and disease-free survival (time to recurrence). We next built a linear classifier model by multivariate Cox regression to predict prognostic outcomes of HCC patients after curative surgery This analysis detected a considerable fraction of variance in HCC prognosis and the area under the ROC curve was about 70%. We further evaluated the model using two other protocols; leave-one-out procedure (n = 264) and independent validation (n = 300). Both were found to have excellent prediction power. The predicted score could separate patients into distinct groups with respect to survival (p-value = 1.8e-12) and disease free survival (p-value = 3.2e-7).

**Conclusion:**

This described model will provide valuable guidance on prognosis after curative surgery for HCC in clinical practice. The adaptive nature allows easy accommodation for future new biomarker inputs, and it may serve as the foundation for future modeling and prediction for HCC prognosis after surgical treatment.

## Background

Hepatocellular carcinoma (HCC) is the fifth most common malignancies in the world, accounting for approximately one million deaths with an increasing trend of new incidences annually [1-3] Surgery is regarded as the one of the standard curative treatments of HCC if the tumor is resectable [4,5]. However, prognosis following surgery differs substantially and such large variation is mostly unexplained. This variation becomes a hurdle in searching for effective and efficacious therapies and cancer management strategies. There is an ongoing search for predictive biomarkers of cancer prognosis, where pathological parameters, protein biomarkers, mRNA expression level, and genomic DNA abnormalities, etc. are surveyed [6-9]. Among these factors, the clinicopathologic parameters are routinely recorded for cancer surgery. Clearly, building a predictive model based on such parameters would be a cost-effective and widely applicable protocol. The most important prognostic endpoints are overall survival (time to death) and disease free survival ([DFS] time to tumor recurrence). There are only a handful of studies identifying tumor size [3,10], staging systems [11] and α-fetoprotein (AFP) [2] in association with these endpoints to date. However, there are no reports on a prediction model systematically built to incorporate all such informative factors. We have surveyed a number of potential predictors to quantify their association with overall survival and DFS. Our primary goal is to develop and validate models that use clinicopathologic parameters and common biomarkers observed at the time of surgery to predict the HCC prognosis. Further, such model must be flexible in accommodating addition factors (e.g. protein biomarkers, gene or microRNA expression signatures) when becoming available.

## Methods

### Study Subjects

In this retrospective study, we analyzed common clinicopathologic data from 600 HCC patients at Queen Mary Hospital, Pokfulam, Hong Kong in the period of 1990 to 2007. These patients were diagnosed with primary HCC and received hepatic surgery as the primary treatment option. Some patients with ≥6 tumor nodules also received surgery and included in this study, given that good liver functions were indicated and the tumors were not near any major vessels and grouped into 1 or 2 clusters. Patients with other malignancies and non-resectable HCC were excluded. Preoperative investigation of the patients included blood biochemistry, alpha-fetoprotein assay, chest x-ray, percutaneous ultrasonography, computed tomography (CT), and hepatic angiography in selected patients. Liver function was assessed by the Child's-Pugh grading. All patients were treated and received the same postoperative care by the same team of surgeons, and had postoperative follow-up every month for the first year, and every 3-6 months thereafter. The minimal duration of follow-up time of the surviving patients was 12 months. Those cases lacking sufficient clinical and follow-up data were not included. Disease-free survival time was calculated from the date of hepatectomy to the date when recurrence was diagnosed.

First, we randomly selected in the training set, 300 patients with available frozen tissue samples (both tumor and adjacent non-tumor) for biomarkers exploration studies by genotyping and mRNA expression profiling. 28 cases that were found missing clinical data or poor sample quality were thus excluded. Table 1 summarizes the demographic and clinicopathologic features of the remaining 272 patients in the training set. The other 300 cases were included in the validation set. There were no significant differences (p-value > 0.05, two-sided tests) in the demographic and clinicopathologic features of HCC patients between the training and validation dataset. The study protocol was approved by the Institutional Review Board of Queen Mary Hospital, and informed consent was obtained from patients regarding to the use of the liver specimens for research.

**Table 1:** Demographic and clinicopathologic characteristics of HCC patients in the initial training set

Variable Name	Mean ± SD/Median or %	Variable Name	Percentage
**Survival (month)**	33.8 ± 29.4/25.3	**Event**	
**Disease free survival (month)**	25.0 ± 28.9/11.7	Deceased	32.2
**Age (year)**	56.0 ± 12.0/56	Censored	67.8
**Male**	80.4%	**Child's grade**	
**Liver Function Parameters**		A	97.3
AFP [log_10_] (ng/mL)	2.18 ± 1.39/2.03	B	2.7
SGPT (U/L)	61.0 ± 51.4/46	**Family History of HCC**	20.7
SGOT (U/L)	64.3 ± 53.9/49	**Smoking**	
BILIRUBIN (μM)	14.5 ± 11.2/12	No	55.6
ALBUMIN (mg/mL)	40.2 ± 4.7/41	Moderate	30.5
		Heavy	13.9
**Tumor Size (cm)**	7.6 ± 4.1/6.5	**Alcohol Drinking**	
**Tumor Recurrence**	51.1%	No	60.5
**Venous Infiltration**		Moderate	23.3
Absence	50.6%	Heavy	16.2
Presence	49.4%	**No. of tumor nodule(s)**	
**Non-tumorous liver histology**		1	76.3
Cirrhotic	57.1%	2	6.8
Non-cirrhotic	14.0%	3	1.5
Chronic hepatitis	28.9%	4	1.1
**pTNM Stage**		5	0.4
I	3.0%	6	0.8
II	41.1%	Multiple >6	13.2
IIIA	35.5%	**Edmondson Grade**	
IV	20.3%	Undifferentiated	1.3
**AJCC Stage**		Poorly Differentiated	18.3
I	41.5%	Moderate Differentiated	59.2
II	27.9%	Well Differentiated	21.3
IIIA	21.9%	**HBsAg Status**	
IIIB	7.5%	Positive	86.1
IV	1.1%	Negative	13.9

* Drink and smoking data were self-reported: moderate drinking < = 2 drinks/per day; heavy drinking >2 drinks/per day; moderate smoking < = 1 pack/day; heavy smoking >1 pack/day

### Clinicopathological Parameter Measurements

The clinicopathological features of the patients analyzed were sex, age, tumor size, number of tumor nodules, cellular differentiation according to the Edmondson classification, venous infiltration without differentiation into portal or hepatic venules, tumor node metastasis stage (pTNM and AJCC), serum hepatitis B surface antigen (HBsAg) status, and background liver disease in nontumorous liver tissue. They were analyzed as we previously described [12]. In addition we also obtained self-reported life-style parameters such as cigarette smoking (moderate smoking: ≤1 pack/day; heavy smoking: >1 pack/day) and alcohol drinking (moderate drinking ≤: drinks/per day; heavy drinking: >2 drinks/per day).

### Statistical Analysis

We examined whether the clinicopathologic phenotypes that were recorded at the time of surgery might predict cancer prognosis. There are a number of statistical learning techniques able to serve as classifier to make predictions. These include linear model, vector machine, neuron networks, and others. However, many of these methods do not directly accommodate two-dimensional outcome (e.g. survival and DFS). Herein, we used the univariate parameter selection and multivariate Cox model classifier, as previously described [7,8].

In brief, we applied Cox regression models to screen the initial training dataset for clinicopathologic parameters associated with survival outcomes. All significant clinicopathologic parameters were included into a multivariate Cox model. The output of this approach is a linear predictor that could serve as the classifier. Clearly, such a model can separate long vs. short survival patients in the training data. The potential for an over-fitting bias was acknowledged by assessing the prediction accuracy of the leave-one-out (LOO) procedure. In parallel, we also evaluated the prediction performance on an independent testing set.

The log rank p-values did not directly reflect the prediction accuracy, because the sample sizes were different in the training and testing datasets. Instead, utilization of the time-dependent ROC and AUC was used to measure prediction performance [13]. In detail, at a given time *t*, we define

where c denotes the cutoff value and T denotes the survival time. By these means, we generated ROC for every time point and calculated the AUC.

## Results

Table 1 summarizes the demographic and pathologic parameters of the 272 patients that were used as the initial training set. Nearly 2/3 of the patients were right-censored (67.8%) and 1/3 (32.2%) of the patients ceased (failure) upon data analysis. Half of the patients (51.1%) suffered from tumor recurrence during the follow-up period. The primary endpoints employed were overall survival (Table 2) and DFS (Table 3). In a simple Cox model, the endpoints were found to be significantly associated with tumor size, serum AFP levels of alpha fetoprotein (AFP), total albumin concentration (ALBU), venous infiltration (VENINV), tumor stage (pTNM and AJCC), and the number of tumor nodule (NOTN). Most notably, pTNM stage and AJCC stage were highly correlated with the primary endpoints. As expected, overall survival was strongly associated with tumor recurrence and since this could not be observed at the time of surgery, it was decided not to include this factor into the prediction model.

**Table 2:** Effect of demographic and clinical parameters on survival outcome in the initial training set (N = 272)

Variable Name	Coefficient*	p-value	Variable Name	Coefficient	p-value
**Poly (age,2)**	-	0.58	**Tumor recurrence**	1.47	5.7e-8
age	-0.04	0.37	**Gender, male**	0.154	0.59
age^2^	0.0004	0.33	**Family history**	-0.082	0.77
**Liver function parameters**			**Smoking**		
AFP [log_10_] (ng/mL)	0.229	0.002	No	-0.464	0.13
SGPT (U/L	-0.003	0.2	Moderate	-0.385	0.24
SGOT (U/L)	0.0022	0.22	Heavy	ref	ref
BILIRUBIN (μM)	-0.011	0.4	**Alcohol drinking**		
ALBUMIN (mg/mL)	-0.042	0.02	No	0.750	0.05
**Venous infiltration**	1.13	8.4e-07	Moderate	0.817	0.05
**Non-tumorous liver histology**		0.628	Heavy	Ref	ref
**Tumor size**	0.0612	0.004	**No. of tumor nodule(s)**	-	
**pTNM stage**			1	ref	ref
I	-16.56	1.0	2	0.822	0.023
II	Ref	Ref	3~6	0.989	0.10
III	1.05	7.0e-05	Multiple >6	0.841	0.0016
IV	1.30	1.4e-05			
**AJCC stage**			**Child's grade, B**	-0.141	0.84
I	Ref	ref			
II	0.78	8.9e-03	**Edmondson grade**	-	0.15
III & IV	1.46	7.0e-08	**HBsAg +**	0.357	0.31

* The regression coefficient in the Cox proportional hazards model. The hazard rate ratio can be calculated as the exponentiation of the regression coefficient.

**Table 3:** Effect of demographic and clinical parameters on disease free survival in the initial training set

Variable Name	Coefficient	p-value	Variable Name	Coefficient	p-value
**Poly (age,2)**	-	0.32	**Gender, male**	0.114	0.605
age	-0.06	0.20	**Family history**	0.304	0.13
age^2^	0.0005	0.16	**Smoking**		
**Liver function parameters**			No	-0.476	0.06
AFP [log_10_] (ng/mL)	0.107	7.5e-5	Moderate	-0.414	0.12
SGPT (U/L	-0.0001	0.91	Heavy	ref	Ref
SGOT (U/L)	0.003	0.02	**Alcohol Drinking**		
BILIRUBIN (μM)	0.002	0.72	No	0.009	0.97
ALBUMIN (mg/mL)	-0.030	0.04	Moderate	0.117	0.67
**Venous Infiltration**	1.06	3.0e-09	Heavy	ref	ref
**Non-tumorous liver histology**		0.97	**No. of tumor nodule(s)**		
**Tumor size**	-0.012	0.04	1	ref	ref
**pTNM Stage**			2	0.611	0.05
I	0.302	0.56	3~6	0.500	0.39
II	Ref	Ref	Multiple >6	0.963	1.1e-5
III	0.924	8.7e-06			
IV	1.20	2.8e-07	**Child's grade, B**	0.359	0.48
**AJCC Stage**					
I	ref	ref	**Edmondson Grade**	-	0.29
II	0.67	2.6e-03	**HBsAg +**	-0.007	0.98
III & IV	1.16	2.1e-08			

After evaluating all variables in Tables 2 &3, we selected those of significant association with survival and DFS into the multiple regression model (proportional hazards regression). This Cox model employed AFP, ALBU, VENINV, tumor size, new AJCC and NOTN to predict HCC prognosis (survival and DFS). The pTNM stage was not included into the model since this is an old staging system and is currently highly correlated with the new AJCC stage in our dataset. Attempts to incorporate pTNM stage into the prediction models had little impact on results.

**Figure 1:**
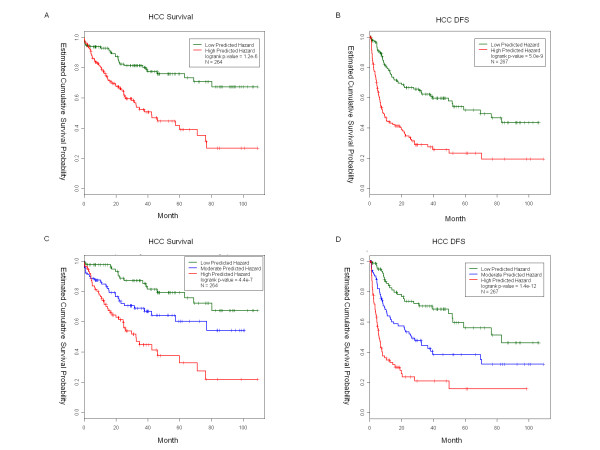
**Kaplan-Meier survival curves of HCC patients in the training set**. Relative hazard (*h*) was predicted for cancer survival (A) and disease-free survival (B) using a leave-one-out procedure. Patients were equally divided into two groups based on *h*, and their Kaplan-Meier survival functions were compared by log-rank test. Alternatively, we divided patients into three equal-sized groups based on their *h*, and observed excellent separation. Such results suggest the prediction is rather robust, and not sensitive to choice of grouping. The vertical bars on the survival curve denote censored patients.

The prediction model performed well on the training data, but was subject to an over-fitting bias from the standpoint of machine-learning. Therefore, the prediction performance was assessed using the leave-one-out (LOO) method. The LOO procedure was performed on 264 patients who have complete survival and covariates data. The analysis consisted of 264 loops. The first step within each loop is to reserve one patient (i.e. *patient*_*i*_) for testing and use the remaining 263 patients to fit a multivariate Cox model (termed as *model*_-*i*_). This approach allows *model*_-*i *_to be independent from *patient*_*i*_. Secondly, the relative hazard (denoted as *h*_*i*_) was predicted for *patient*_*i *_based on his/her covariates and *model*_- _Upon completion of the 264 loops, *h*_*i *_for every patient is obtained. Lastly, equal division of the testing samples into two groups based on *h*_*i*_, was followed by evaluating the prediction accuracy using a Kaplan Meier plot and log rank test. The LOO procedure was also conducted for DFS with 267 patients. The Cox model based on clinicopathologic parameters offered substantial prediction power as shown in Figure 1. The high and low-risk groups were significantly different in terms of survival and DFS (log rank p-values of 1.2e-6 and 5.0e-9, respectively). About 80% patients in the predicted low-risk group survived over 60 months after surgery. Conversely, only about 40% patients in the high-risk group survived 60 months (Figure 1A). To further demonstrate robustness of the data, the patients were divided into three equal sized groups according to *h*_*i *_(Figures 1C and 1D). The results of this additional grouping further demonstrate separation in terms of survival and DFS.

**Figure 2:**
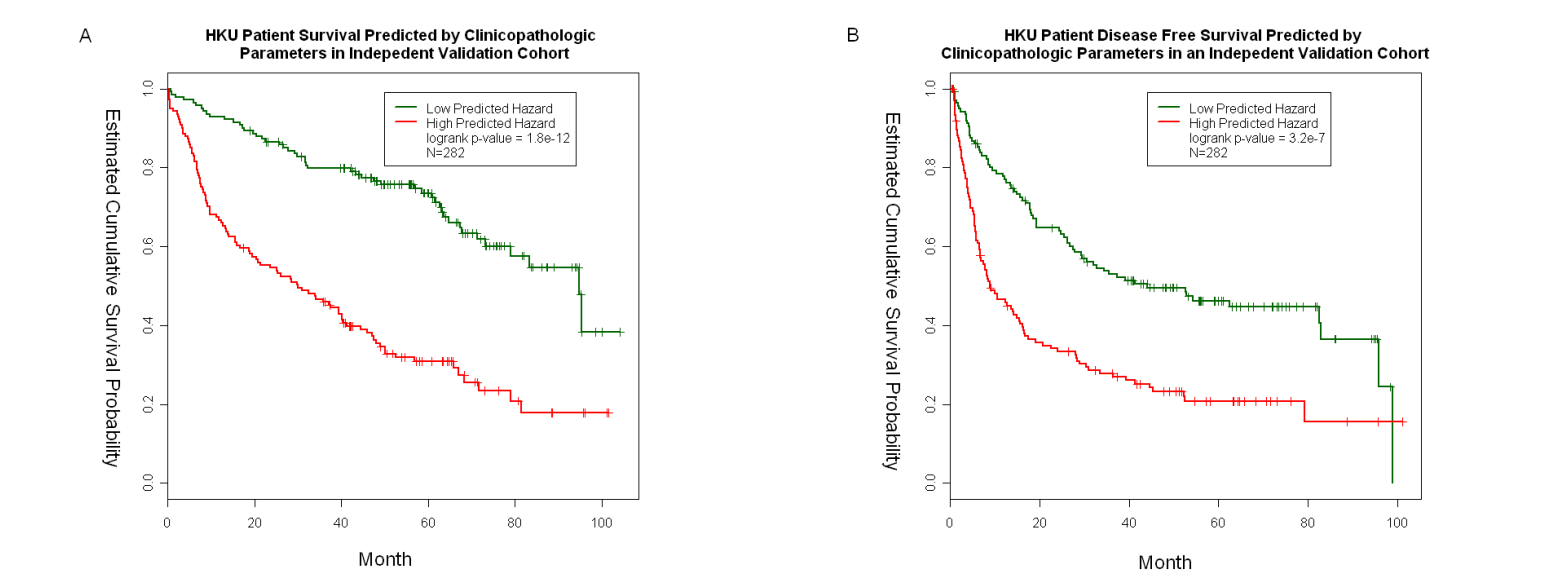
**Kaplan-Meier survival curves of HCC patients in the validation set**. We first fit a multivariate Cox model using the initial training set focusing on overall cancer survival (A) and disease-free survival (B). This model was used to predict the relative hazard (*h*) for an independent testing set. Next, the testing patients were equally divided into two groups based on predicted *h*, and their Kaplan-Meier survival functions were compared by log-rank test.

Motivated by the significant findings in the initial training dataset, we further tested this model independently in the validation set of separate 300 patients. This test set consisted of 272 patients who had complete outcome and covariate information. Analysis of both the training and testing samples clearly show similar results in terms of survival, DFS (see Additional file 1) and covariates distribution (see Additional file 2). The selected covariates were significantly associated with cancer prognosis in the testing set with respect to overall survival (Table 4) and disease-free survival (Table 5). As a result of this association, it was concluded that the variable distributions in the testing patients were similar to the training patients and are therefore suitable for independent validation. The entire training data set was used to fit a multivariate Cox model and then employed to predict the relative hazard for the testing samples. The next step involved equally divided the testing samples into two groups based on predicted hazard followed by a log rank test (Figure 2). The predicted high and low-risk groups were significantly different in survival (log-rank p-value = 1.8e-12) and DFS (log-rank p-value = 3.2e-7).

**Table 4:** Effect of clinical parameters on survival outcome in the testing set

Variable Name	Coefficient*	p-value	Variable Name	Coefficient	p-value
**AFP [log_10_]**	0.194	7.3e-04	**AJCC Stage**		
**ALBUMIN**	-0.040	0.02	I	Ref	-
**Tumor size**	0.061	1.2e-4	II	0.92	3.4e-03
**pTNM Stage**			III & IV	1.68	8.0e-15
I	-0.65	0.15	**No. of tumor nodule(s)**		
II	ref	-	1	Ref	-
III	1.58	2.3e-04	2	0.81	7.7e-04
IV	2.27	1.8e-07	3~6	0.90	2.2e-03
**Venous Infiltration**	0.86	2.3e-07	Multiple >3	1.08	2.8e-05

* The regression coefficient in the Cox proportional hazards model. The hazard rate ratio can be calculated as the exponentiation of the regression coefficient.

**Table 5:** Effect of clinical parameters on disease free survival in the validation cohort

Variable Name	Coefficient	p-value	Variable Name	Coefficient	p-value
**AFP [log_10_]**	0.152	4.0e-03	**AJCC Stage**		
**ALBUMIN**	-0.020	0.22	I	ref	-
**Tumor size**	0.067	7.0e-6	II	0.66	4.9e-04
**pTNM Stage**			III & IV	1.24	5.1e-11
I	-0.05	0.87	**No. of tumor nodule(s)**		
II	ref	-	1	ref	-
III	0.74	8.1e-03	2	0.64	5.5e-03
IV	1.34	3.5e-06	3~6	1.33	1.3e-06
**Venous Infiltration**	0.60	5.1e-05	Multiple >6	0.97	9.2e-05

* The regression coefficient in the Cox proportional hazards model. The hazard rate ratio can be calculated as the exponentiation of the regression coefficient.

**Figure 3:**
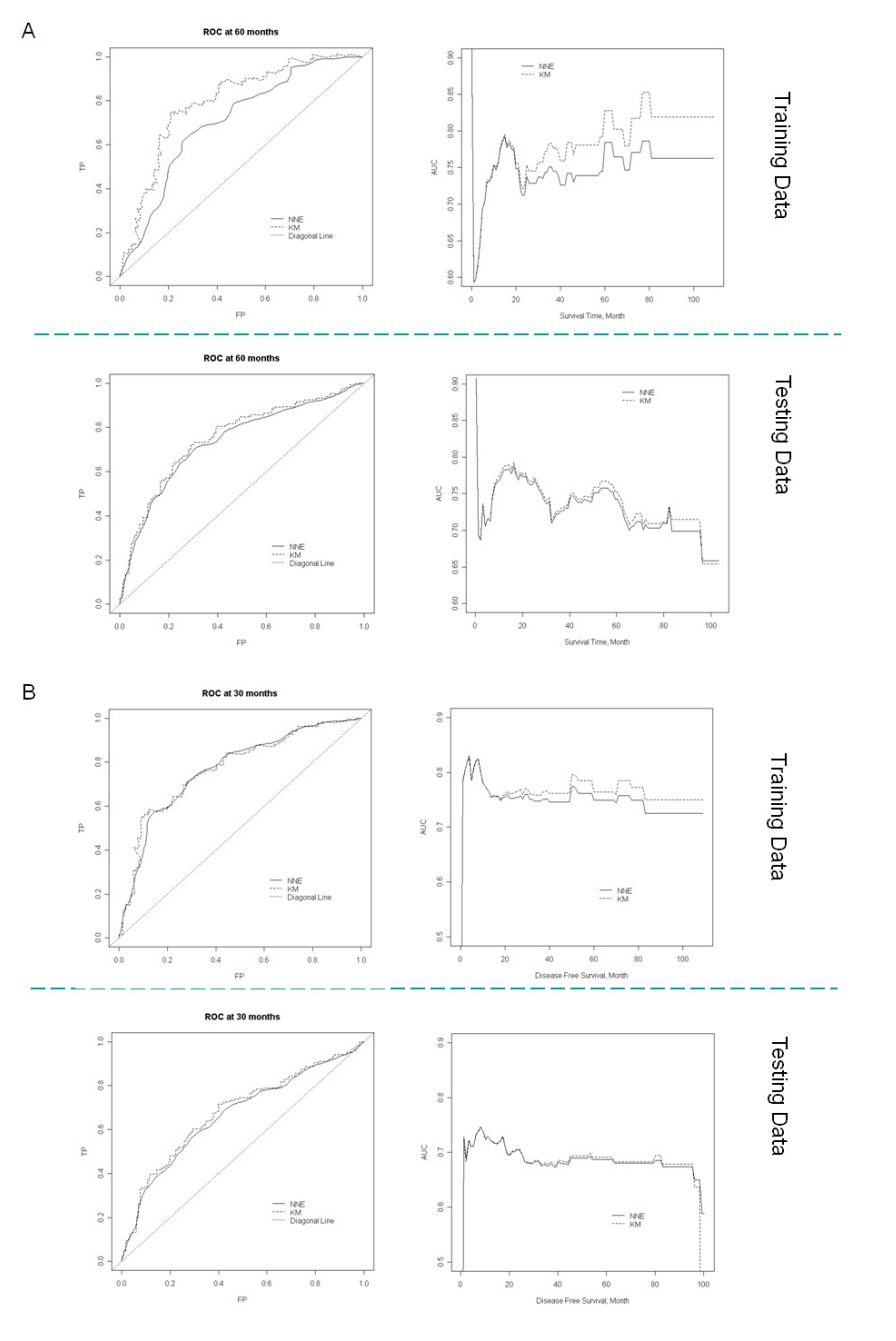
**ROC curve analyses of HCC patients in the training and testing datasets**. Multivariate Cox model built on the initial training set was used to predict cancer prognosis in the training set and testing set. Time-dependent ROC and AUC were computed in the intervals of (A) 60 months and (B) 30 months to quantify the prediction accuracy.

Lastly, the time-dependent ROC curves (in Figure 3A, *t *= 60 months for survival; in Figure 3B, *t *= 30 months for DFS) were derived along with the area under the curve (AUC) for all time points. The average AUC was 0.7 for the testing set, indicating considerable predicting power. The AUC was slightly higher (0.75) in the training dataset, reflecting the over-fitting bias.

## Discussion

The purpose of our study was to develop a systematic model according to universally recognized clinicopathologic parameters for improved accuracy of prognostic outcome prediction in HCC patients after curative surgery. Therefore, all acknowledged factors associated with outcomes were evaluated. Notably, certain important parameters, such as tumor staging and venous infiltration status, would require postoperative histological examination of the resected tissues or biopsy samples of the patients, unless future radiological examination using dynamic MRI could provide such definitive diagnosis. Nevertheless our primary contribution is the modeling framework which incorporates multiple parameters in prediction prognosis. Its flexible nature allows us to easily remove parameters (e.g. VENINV when either biopsy samples are not available or diagnostic radiography data is not affirmative) or to add new biomarkers (e.g. newly identified gene signatures). Several studies of HCC have reported the ability to use clinicopathologic parameters or biomarkers towards grouping subjects and predicting survival outcomes [2,3,14]. However, most studies were small or without an independent validation set. Likewise, there are no models built to systematically incorporate all factors to predict outcome. In addition, the existing prediction rules appear to be *ad hoc*, involve multiple arbitrary cutoffs and lack statistical rigor. Lastly, the performance of such rules has not been formally assessed by such analyses, e.g., ROC.

The salient contribution of this publication is the identification of certain common clinicopathologic parameters and biomarkers strongly associated with the prognostic outcome that are further confirmed by two different protocols: LOO and independent validation. Previous studies have revealed some of these predictors, but often lack reproducibility which may due to the difference among patient populations. For example, the majority of the Chinese patients carry hepatitis B [15], while hepatitis C virus is prevalent in Western Europe [16]. The relatively small sample sizes of the aforementioned data sets contribute to the inconsistent results. Herein, we provide confirmation to a previous report using a large sample size and validation data. The first group of predictors highlighted in this paper is the tumor stage. There are growing numbers of staging systems available but unfortunately not one is perfect with each having individual strengths and weakness. Conversely, our data sets show staging systems are highly correlated. The following parameters were found to be strongly associated with prognosis and prediction value: AFP, albumin level, venous infiltration and the number of tumor nodules. Among these predictors, the serum albumin level may reflect the conditions of patients liver physiology, while the rest of other factors hint to the biology or characteristics of the HCC tumor per se. This may reflect the importance of both the host (or the microenvironment) and tumor factors contributing to the outcomes. The self-reported variable alcohol consumption was marginally significant in a univariate model but was not used and determined to be non-significant in our multivariate Cox model. This finding is highly consistent with previous report on Chinese HCC patients [15,17] where dichotomized serum albumin and tumor size, number of tumor nodules, venous infiltration, and tumor stage were significantly associated with cancer prognosis after surgery.

The prediction power of the predicted hazard (*h*) was demonstrated in this report, which is literally the linear combination of many predictors. A number of studies were conducted quantifying the predictive power of clinicopathological parameters, and our results are consistent with previous reports on vascular invasion [18,19], AFP level [15,19] and tumor size [19] in term of hazard ratios. However, this study did not measure some variable, e.g., portal hypertension and bilirubin [20], therefore, we could not directly assess their predictive value. 97.3% of our patients were classified as Child-Pugh grade A, and we found Child-Pugh grade not to be a significant predictor (possibly due to lack of statistical power). Nevertheless, our primary goal is to develop the objective and flexible framework, which can easily accommodate additional biomarkers when becoming available. There are well known scoring systems to classify HCC, including Child-Pugh [18], the Oku [11], Advanced Liver Cancer Prognostic System (ALCPS, applicable to patients with advanced HCC who were not amendable to surgery or locoregional therapy) [21], and Barcelona Clinic Liver Cancer Group [20,22]. As the drawback, these scores have been devised by a series of ad hoc rules. In the other hand, predicted hazard is objective and can readily incorporate a new patient and biomarkers information. This can be simply performed by updating the linear model and the set of coefficients and will allow us to continuously update the model when new data becomes available. This is particularly important as new biomarkers based on molecular studies of HCC could be incorporated into the model. HCC has a heterogeneous etiology and many factors (e.g. patients' ethnicity and genetic background) may affect prognosis. Therefore, this model may not be directly applicable to a different HCC cohort in the Western countries. This approach should serve as a general framework, where the Cox linear classifier can be trained on a particular cohort and applied to future patients.

A considerable fraction of our HCC patients achieved 5+ years of DFS after surgery, especially for those with favorable clinicopathologic profiles as highlighted in our previous reports [15]. The cutoff of 5 or 10 years is widely used to define the cure of HCC, however, such thresholds are arbitrary. The categorization of a patient with 5 year of DFS as a cure, while classifying another patient with 4.9 year DFS as a failure does not lead to valid conclusions. Therefore, treatment of survival and DFS as quantitative traits was performed without the application of any cutoffs or subjective dichotomization of the clinicopathologic parameters. HCC patient follow-up is a non-trivial task given the ever increasing mobility of an urban population. Due to improved treatment and management, HCC patients live longer and thus make follow-up evaluations a challenge. One strategy is to only analyze the patients with observed events [15,23], but this approach would greatly reduce the sample size (as well as statistical power), since many of the data point were censored (Figure 1). Instead, we employed of time-to-event methods (e.g. Cox model), where the censored data also contributed to the test and improved statistical power. Moreover, the Cox model also quantifies the strength of the association between clinicopathologic parameters and prognosis (in the form of relative hazard), which is actually the foundation for prediction.

In the past decade we have observed a fast growth of literature using gene expression profiles and DNA abnormality to predict cancer prognosis. However, these approaches have not achieved real clinicopathologic applicability because of the inter-institutional variation caused by numerous factors such as; array platform, statistical algorithm, reagent, laboratory condition/protocol. In contrast, clinicopathologic parameters are more standard, robust, and available worldwide. Models built on clinicopathologic factors can be more readily used in today's hospital practice. Further, the linear model should certainly include new biomarkers (to improve prediction power if there is conclusive evidence that a biomarker gives additional information conditioning on known factors. Recent studies [24,25] revealed gene expression in tumor and adjacent normal liver tissues (suggesting a so called "field-effect") were predictive for HCC prognosis. It has been suggested that mechanistically these signatures capture tumor status, damage to liver tissue and the state of inflammation which relates to the likelihood of subsequent tumors arising [23,25]. However, all such studies failed to incorporate clinicopathological and expressional predictors together. In such cases, the identified expressional predictors first capture the same information as clinicopathological parameters (e.g. cancer stage), and the performance for the expressional biomarkers would not necessarily outperform clinicopathological predictors (unpublished results). Herein, we argue that in order to capture information from gene expression that was not redundant to clinicopathologic data, the clinicopathologic parameters would be included during the search for expression signatures, and our hazard score model will serve for this purpose.

In summary, we have retrospectively analyzed the association of common clinicopathologic parameters with clinical outcomes in a large sample cohort (n = 572) of HCC patients after curative surgery. We quantified the strength of the association and then built a classifier based on multi-variates Cox regression. This approach has excellent prediction power. Nonetheless, there is still a large fraction of variance that has not been explained since the area under the ROC is around 70% in validation data. The search for other biomarker to improve the prediction of HCC survival will certainly be an important direction for future research. As a caveat, the models that employ clinicopathologic parameters are often competitive with most of the models incorporating gene expression data. This is due to both set of predictors capturing the tumor stage [8]. Hence, the model described in this paper should serve as a foundation for future work when biomarkers at the protein, mRNA or DNA level are considered. Furthermore, the selection of additional biomarkers should be based on common clinicopathologic parameters; otherwise, they may recapture similar information and provide little extra predictive value.

## Conclusion

The present study described a Cox linear regression model based on common clinicopathologic parameters, which provides valuable guidance on prognosis after curative surgery for primary HCC among Chinese patients in clinical practice. The adaptive nature allows easy accommodation for future new biomarker inputs, and it may serve as the foundation for future modeling and prediction for HCC prognosis after surgical treatment.

## Competing interests

KH, CZ, MDF, JRL, MM and HD engaged in this work as employees of Merck & Co., Inc., a leading pharmaceutical company; other than that, all other authors disclose no competing interest.

## Authors' contributions

JL and RP contributed to the study design and conception. NL contributed in study execution. IN helped in the pathological definition. RP was responsible for acquisition of clinical samples and gave advices on samples' clinical background. PS provided statistical advice and critique. JL and RP carried out critical revision of the manuscript. KH conducted the analysis. MM, CZ, MF, JL, HD and RP provided critical review on the manuscript. The manuscript was drafted and written by KH and JL. MM, CZ, MF, JL, HD and RP provided critical review on the manuscript. All the authors approved this manuscript.

## Pre-publication history

The pre-publication history for this paper can be accessed here:

http://www.biomedcentral.com/1471-2407/9/389/prepub

## Supplementary Material

Additional file 1 **Clinical outcomes of HCC patients from the training and validation set**. The distribution of overall survival rate (A) and disease-free survival rate DFS (B) reveals no significant difference among the HCC patients between the training and validation samples. Click here for file

Additional file 2 **Characteristics of validation dataset (N = 300)**. The demographic and clinicopathologic parameters of HCC patients in the validation set show no significant difference from the initial training set. Click here for file
