# Supplementary figures and images for: Predicting prognosis in hepatocellular carcinoma after curative surgery with common clinicopathologic parameters
Source: BMC Cancer. 2009 Nov 3;9:389. doi: 10.1186/1471-2407-9-389 (PMC2785835; doi:10.1186/1471-2407-9-389)

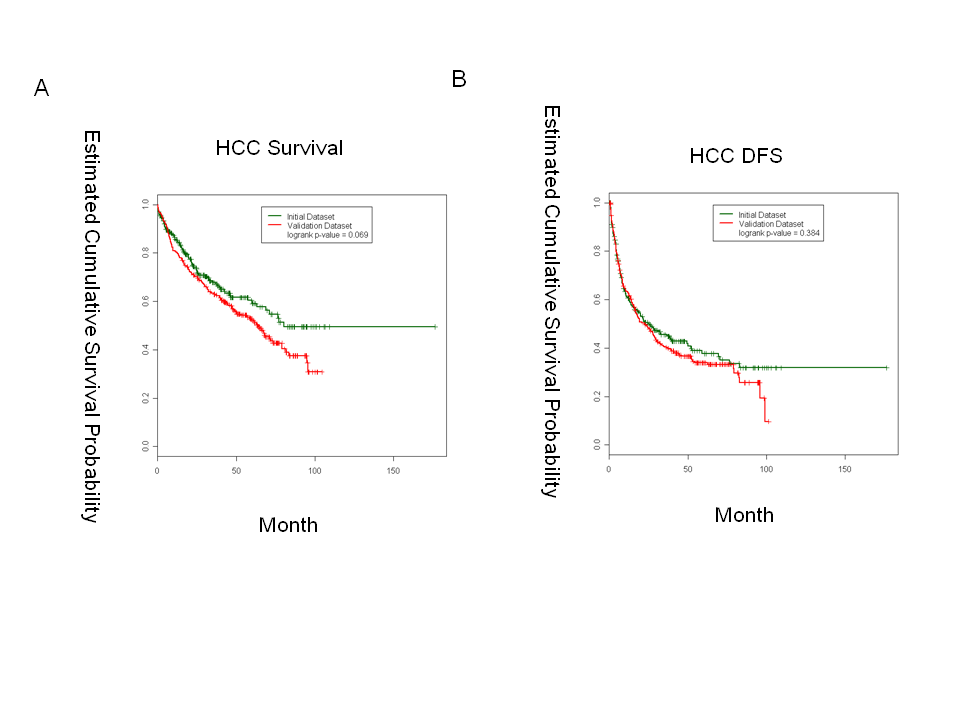

Supplement: Additional file 1 — Clinical outcomes of HCC patients from the training and validation set. The distribution of overall survival rate (A) and disease-free survival rate DFS (B) reveals no significant difference among the HCC patients between the training and validation samples. [file 1471-2407-9-389-S1.tiff]
