# Supplementary material for: Predicting prognosis in hepatocellular carcinoma after curative surgery with common clinicopathologic parameters
Source: BMC Cancer. 2009 Nov 3;9:389. doi: 10.1186/1471-2407-9-389 (PMC2785835; doi:10.1186/1471-2407-9-389)
Supplement: Additional file 2 — Characteristics of validation dataset (N = 300). The demographic and clinicopathologic parameters of HCC patients in the validation set show no significant difference from the initial training set. [file 1471-2407-9-389-S2.doc]

**Additional Table S1: Characteristics of validation dataset (N = 300)**

| **Variable Name** | **Mean ± SD / Median or %** |  | **Variable Name** | **Percentage** |  |
| --- | --- | --- | --- | --- | --- |
| **Survival (month)** | 43.5 ± 28.2 / 43.9 |  | **AJCC Stage** | - |  |
| **Disease free survival (month)** | 28.6 ± 27.7 / 16.8 |  | I | 37.0 |  |
| **AFP [log_10_] (ng/mL)** | 2.2 ± 1.4 / 2.0 |  | II | 30.7 |  |
| **ALBUMIN (mg/mL)** | 39.4 ± 4.4 / 40 |  | IIIA | 13.7 |  |
| **Tumor size (cm)** | 6.6 ± 4.8 / 5.0 |  | IIIB | 13.0 |  |
| **pTNM Stage** |  |  | IV | 0.7 |  |
| I | 10.0 |  | **Tumor Nodule** | - |  |
| II | 29.3 |  | 1 | 77.0 |  |
| IIIA | 33.3 |  | 2 | 9.7 |  |
| IIIB | 0.7 |  | 3 | 3.7 |  |
| IVA | 21.3 |  | 4 | 1.3 |  |
| IVB | 0.7 |  | 5 | 0.7 |  |
| **Venous Infiltration** |  |  | Multiple >6 | 7.7 |  |
| Absence | 50.3 |  |  |  |  |
| Presence | 49.7 |  |  |  |  |
